# ASSESSING THE NEW INFLATION REDUCTION ACT’S IMPACT ON EXPANDING MEDICARE PART D LOW-INCOME SUBSIDIES

**DOI:** 10.1093/geroni/igac059.2896

**Published:** 2022-12-20

**Authors:** Feng-Hua Loh, Bruce Stuart

**Affiliations:** Touro College of Pharmacy, New York, New York, United States; University of Maryland School of Pharmacy, Baltimore, Maryland, United States

## Abstract

The recently enacted Inflation Reduction Act (IRA) of 2022 extends full low-income subsidies (LIS) to Medicare Part D recipients with limited assets and incomes up to 150% of the Federal Poverty level (FPL) beginning in 2024. Under current law, full Part D subsidies (no annual premiums and nominal cost sharing) are available only to those with incomes up to 135% of the FPL. Partial subsidies are available to those with incomes between 135% and 150% of FPL. Increasing the level of benefits to those currently only eligible for partial subsidies has the potential of significantly lowering out-of-pocket medication costs that pose a barrier to adherence for many low-income Medicare beneficiaries, but the outcome of the new policy will depend on how many enroll. Historically, enrollment among those eligible for partial subsidies has hovered around 30%, but the latest data on LIS participation rates are for 2014. We undertook this study to update LIS eligibility and enrollment numbers to 2019, with a particular emphasis on identifying characteristics of beneficiaries who may benefit from the new policy. Using data from the Medicare Current Beneficiary Survey, we determined that 73% of beneficiaries eligible for full subsidies under current law were enrolled, but only 25% eligible for partial subsidies were enrolled. We conclude that while the IRA will raise the overall level of Part D subsidies, it will be important to engage stakeholders and promote outreach to maximize participation in the leadup to implementation.

